# Inhibitory effect of vitamin C on *Aspergillus parasiticus* growth and aflatoxin gene expression

**DOI:** 10.18502/cmm.4.3.170

**Published:** 2018-09

**Authors:** Maryam Akbari Dana, Parivash Kordbacheh, Roshanak Daei Ghazvini, Maryam Moazeni, Ladan Nazemi, Sasan Rezaie

**Affiliations:** 1 Division of Molecular Biology, Department of Medical Mycology and Parasitology, School of Public Health, Tehran University of Medical Sciences, Tehran, Iran; 2 Invasive Fungi Research Centre, Department of Medical Mycology and Parasitology, School of Medicine, Mazandaran University of Medical Sciences, Sari, Iran; 3 Department of Medical Biotechnology, School of Advanced Technologies in Medicine, Tehran University of Medical Sciences, Tehran, Iran

**Keywords:** Aflatoxin, aflR gene, Aspergillus parasiticus, Gene expression, Vitamin C

## Abstract

**Background and Purpose:**

Aflatoxin is known as one of the most important mycotoxins threatening human life. This toxin is produced by *Aspergillus* species, which is the common cause of agricultural product contamination. The use of organic compounds has been always considered for the inhibition of fungal growth and toxin production. Regarding this, the aim of the present study was to investigate the effect of vitamin C on the rate of fungal growth, *aflR* gene expression, and toxin production.

**Materials and Methods:**

For the purpose of the study, first, *Aspergillus parasiticus *ATCC15517 was cultured in Sabouraud dextrose agar medium containing vitamin C at concentrations of 200, 100, 50, 25, 12.5, 6.25, and 3.1 mg/ml and temperature of 28°C for 72 h. Then, the amount of aflatoxin produced in the presence of vitamin C was measured through high performance liquid chromatography. Finally, by extracting the DNA of the cultured samples, the *aflR* gene expression level was evaluated by means of real-time polymerase chain reaction at different concentrations of vitamin C.

**Results:**

The results showed that mycelium deformation was started at the vitamin C concentration of 50 mg/ml, and that only fungal spores were observed at higher concentrations. The levels of total aflatoxin and its subsets, namely B_1_, B_2_, G_1_, and G_2_, in the presence of vitamin C were estimated as 5.9, 1.9, 0.2, 3.5, and 0.3 ppm, respectively. On the other hand, these values were respectively obtained as 207.5, 73.6, 4.5, 123.4, and 6 ppm in the absence of vitamin C. Measurement of the expression level of *aflR* genes showed that the level of gene expression decreased to 68% and up to 81% at the vitamin C concentrations of 25 and 50 mg/ml, respectively.

**Conclusion:**

This study showed that vitamin C, as a human-compatible compound, could be considered a good agent to protect agricultural products against fungal aflatoxin.

## Introduction

Diseases caused by the contamination of food with mycotoxins have always been considered as an important challenge in the clinical field due to their high mortality rate and therapeutic costs [1]. Aflatoxin is one of the most known mycotoxins in the field of agricultural product contamination. This toxin is produced by *Aspergillus* family, especially two species of *A. Flavus *and *A. Parasithicus*, which are the most prevalent causes of the fungal contamination of agricultural products [2, 3].

Aflatoxin produces a wide variety of diseases due to its carcinogenic, mutagenic, and immunosuppressive properties and accounts for relatively high morbidity and mortality rates in humans [4, 5]. Genetic analysis of *Aspergillus* fungi is indicative of the regulatory role of *aflR* and *aflJ* genes in aflatoxin biosynthesis. Therefore, mutation in these genes can be considered an appropriate target for the elimination of toxin [6-8]. The process of toxin removal from agricultural products should be monitored for preventing any harms to human beings [9].

Ascorbic acid, known as vitamin C, has been always considered as a good candidate for interventional studies. Due to the presence of this acid in human bodies, it has an increased clinical significance in therapeutic investigations against bacteria, viruses, and fungi [10, 11]. With this background in mind, the present study was conducted to evaluate the effect of ascorbic acid on the growth of *Aspergillus parasithicos*, *aflR* gene expression, and toxin production.

## Materials and Methods

Minimum inhibitory concentration (MIC) was determined by culturing the standard sample. Molecular analysis of *aflR* gene was accomplished using quantitative real-time polymerase chain reaction (RT-PCR). In addition, high-performance liquid chromatography (HPLC) was employed to analyze aflatoxin production.

***2-1. Strain collection***

*Aspergillus parasiticus* ATCC15517 (standard strain) was cultured on Sabouraud dextrose agar medium (Merck KGaA, Germany) at 28°C for 3 days.

***2-2. Antifungal susceptibility testing***

The MIC values of the samples were investigated in the presence of vitamin C according to the Clinical and Laboratory Standards Institute M38-A2 guidelines (2008) [12]. For this purpose, fungal suspensions were prepared from cultures using distilled water. Then, the optical density (OD) of the suspensions were examined by spectrophotometry at 530 nm. The suspensions with an OD range of 0.09-0.13 were used in the study. A suspension was made by a 1:50 dilution of the stock suspension with the RPMI medium, which resulted in a colony count of 0.4×10^4^ to 5×10^4^ CFU/ml.

Vitamin C powder (Osve Pharmaceutical Company, Iran) was used to produce vitamin C solution at a concentration of 400 mg/ml. Following the dilution, vitamin C solutions were prepared at the concentrations of 200, 100, 50, 25, 12.5, 6.25, and 3.1 mg/ml, and then dispensed into a 96-well microdilution plate. At the end of this step, 100 µl of working suspension was added into the microdilution plate. To investigate the effect of vitamin C on fungal growth, the plate was kept at 28°C for 48-72 h, and the turbidity was determined. Positive and negative controls were also utilized in order to test the validity of the test.

***2-3. Aflatoxin extraction using high-performance liquid chromatography***

The next step involved the measurement of the amount of toxin produced by the fungus in the presence of vitamin C. To this end, fungal suspension was cultured at a concentration of 4×10^4^ in potato dextrose broth (PDB) medium containing vitamin C. In addition, two PDB media were employed as positive and negative controls, one of which contained 50 ppm pure aflatoxin (standard sample) and the other one had no fungal suspension, respectively.

The samples were stored at 35°C for 10 days. To extract aflatoxin from the PDB medium, HPLC immunoaffinity columns were used. Furthermore, 80% methanol buffer containing extra pure NaCl (Merck, Germany) was used as a buffer to extract aflatoxin from the media in a chromatographic column. Extracellular PDB media were removed from the samples by means of a glass-fiber filter paper. Then, the column was washed with phosphate buffered saline and balanced with the working buffer. Finally, toxin separation was carried out by applying 50 ml of the samples to the column [12].

***2.4. RNA extraction***

This study was targeted toward the investigation of the effect of vitamin C on the level of *aflR* gene expression. To this end, *A. parasiticus* ATCC15517 was cultivated in an RPMI medium containing vitamin C at the concentrations of 25 and 50 mg/ml for 3 days at 28°C in order to extract RNA.

The mycelium collected from the fungal cultures was washed twice with 1XPBS, and then centrifuged at 1200× g for 5 min. In the next stage, the samples were flash-frozen in liquid nitrogen, and then grinded to a fine powder. The mycelial powder was suspended to goanidine isothiocyanate (4 M, 2% beta-mercaptoethanol, and 1% sodium lauroyl sarcosine in final concentration) and then homogenized. Subsequently, sodium acetate was added in 1/10 volume of suspension. Phenol and chloroform were then added at 1:1 and 1:5 ratios, respectively. RNA was extracted by centrifugation at 1200× g for 10 min. In the next stage, genomic DNA was removed by using DNase kit (Fermentas, USA), and complementary DNA was synthesized using the Prime Script RT kit (Fermentase, USA).

***2.5. Real-time polymerase chain reaction***

In the present study, RT-PCR was used to screen *aflR* gene expression. The β-actin gene (i.e., endogenous reference gene) was used for gene normalization. The primers used for testing the above-mentioned gene in the RT-PCR were designed using Gene Ranger software (Table 1). The quantitative RT-PCR method was performed using the Applied Biosystems StepOnePlus (ABI, USA). The process of quantitative PCR included an initial denaturation step for 30 sec at 95°C, followed by denaturation for 5 sec at 95°C, and then annealing and extension for 30 sec at 60°C.

***2.6. Statistical analysis***

All data analyses were performed in SPSS software for Windows (version 16), using the t-test and Fisher’s exact test. *P-value* less than 0.05 was considered statistically significant.

**Table 1:** Primers used for real-time polymerase chain reaction

***SENSaflR***	5'- CggAACAgggACTTC CggCg - 3'
***Anti*** **-** ***SENS aflR***	5'-gggTggCgggggACTCTgAt -3'
***SENS*** ***βActin***	5'-ACgg TAT TTCCA ACTgggACg-3'
***Anti-SENS ßActin***	5'-TggAgCTTCggTCAACAAAACTgg-3'

## Results

***3.1. Effect of different concentrations of vitamin C on Aspergillus parasiticus growth***

**Figure 1:**
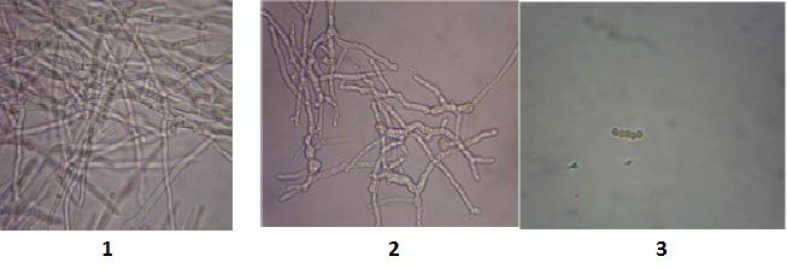
Effect of vitamin C on the growth rate of fungal suspension; 1) concentrations of 25 mg/ml, 2) concentration of 50 mg/ml, and 3) concentration of 100 mg/ml (As seen in the picture, the amount of fungal mass decreases with the enhancement of vitamin C concentration.)

In order to evaluate the effect of vitamin C on fungal growth, fungal suspensions were cultivated in the presence of different concentrations of vitamin C. Then, the samples were subjected to microscopic examination. The results showed that the process of mycelium deformation was started at the concentration of 50 mg/ml, and that only fungal spores were observed at the concentration of ≤ 100 mg/ml (Figure 1).

***3.2. Effect of vitamin C on aflatoxin production***

The HPLC method was used to evaluate the effect of vitamin C on the rate of aflatoxin production. According to the results, in the positive control, the levels of total aflatoxin and its subtypes, namely B_1_, B_2_, G_1_, and G_2_, were obtained as 207.5, 73.6, 4.5, 123.4, and 6.0 ppm, respectively. These values were respectively estimated as 5.90, 1.9, 0.2, 3.5, and 0.3 ppm in the culture containing vitamin C. No aflatoxin was extracted in the negative control. Table 2 tabulates the levels of total aflatoxin and its subsets in the cultured samples containing vitamin C, as well as positive and negative controls.

Based on the findings, culturing fungal suspension with vitamin C resulted in a significant reduction in the amount of aflatoxin, compared to the positive control (containing culture of fungal suspension without vitamin C). It should be noted that in this study, all experiments were repeated three times.

***3.3. Effect of vitamin C on afIR gene expression***

**Figure 2:**
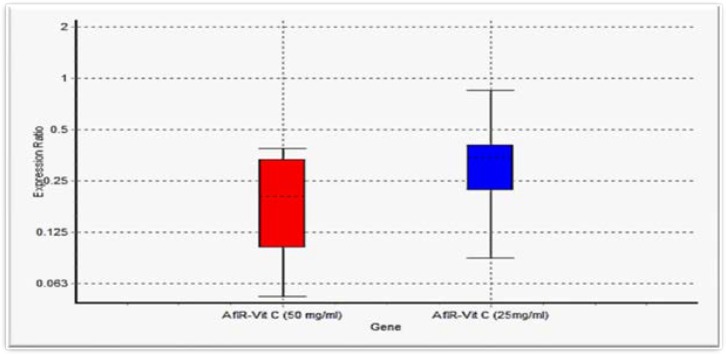
Comparison of *afIR* gene expression at different dilutions of vitamin C

To investigate the effect of vitamin C on *afIR* gene expression, after culturing fungal suspension in the medium containing vitamin C, RNA extraction was carried out according to the protocols mentioned above. Agarose gel electrophoresis experiment confirmed the accuracy and integrity of RNA extraction. Then, RT-PCR was performed using SYBR Green method. The obtained melt curves showed the specificity of the primers. The results of this study demonstrated that gene expression decreased to 68.7% and up to 81% at the concentrations of 25 and 50 mg/ml, respectively (Figure 2).

**Table 2:** Levels of total aflatoxin and its subtypes of B_1_, B_2_, G_1_, and G_2_ in the research samples in the presence of vitamin C and positive and negative controls

	***Total Afla*** ***ppm***	***Afla B1*** ***ppm***	***Afla B2 *** ***ppm***	***Afla G1*** ***ppm***	***Afla G2*** ***ppm***
**Sample**	5.9	1.90	0.2	3.5	0.3
**Positive control**	207.5	73.6	4.5	123.4	6.0
**Negative control**	0.00	0.00	0.00	0.00	0.00

## Discussion

Aflatoxicosis has always been considered an important factor threatening human life. The elimination or reduction of the growth of the fungus generating this toxin and inactivation of toxin in agricultural products are regarded appropriate options to protect these products against fungal aflatoxin [13-15]. There are multiple studies investigating this domain. Verma et al. examined the effect of vitamin A on *A. parasiticus* and demonstrated that this vitamin can reduce the rate of aflatoxin production in this fungus [16].

In another study, Bluma et al. investigated the effect of thyme and clove oil on controlling the contamination of food products with fungal toxin. They showed that clove reduced the aflatoxin production, while mountain rhizome and eucalyptus inhibited the production of this toxin by about 85-90% at all concentrations [17]. Furthermore, Yoshinari et al. examined the effect of *Streptomyces* compounds on the production of aflatoxin and reported that these compounds inhibited the production of this fungal toxin at a concentration of 4 mM [18]

Another study involved the investigation of the inhibitory effect of the methanol extract of Korean soybean paste on mold growth and aflatoxin production in a toxigenic strain of *A. parasiticus* ATCC 15517 using different concentrations of the extract in a yeast-extract sucrose broth. In the mentioned study, the enhancement of the extract concentration resulted in the inhibition of mold growth. However, the inhibition of aflatoxin production was a more remarkable effect in the mentioned study. As a result of extract addition, mycelial weight and aflatoxin production were observed to range from 1.5% to 12.9% and 14.3% to 41.7% by HPLC, respectively [19].

In another study, the inhibitory effect of green onion produced in Korea was investigated on the growth of *A. parasiticus*, a toxigenic strain, using different concentrations of freeze-dried green onion in a solid culture with potato dextrose agar. The mentioned study revealed that this plant could effectively inhibit the growth of *A. parasiticus* [20].

The present study was targeted toward the determination of the effect of vitamin C on fungal growth and expression of genes involved in the biosynthesis of toxin. The statistical results showed that almost 100% of fungal growth stopped when cultured in the medium containing vitamin C at a concentration of 100 mg/ml. On the other hand, the expression of the gene involved in the biosynthesis of toxin decreased to 68% and 81% at vitamin C concentrations of 25 and 50 mg, respectively. 

Accordingly, the research results showed that vitamin C had a significant effect on fungal growth reduction and toxin production (*P<0.01*). Based on the results of the present study, vitamin C can be considered as a good agent to protect agricultural products against fungal contamination and reduce the associated damage due to its adaptation with human body.

## Conclusion

The findings of present study showed that vitamin C, as a human-compatible substance, can interfere with the pathogenesis of *A. parasiticus *by reducing the expression of genes involved in the production of aflatoxin. Given the importance of agricultural products, vitamin C can be used to protect these products against aflatoxin.

## Financial considerations

This research was financially supported by a grant given by Tehran University of Medical Sciences, Tehran, Iran.
